# Malaria and global warming in perspective?

**DOI:** 10.3201/eid0604.000431

**Published:** 2000

**Authors:** P Reiter


*Salmonella* infections acquired in other countries are frequently diagnosed in travelers recently returned to the United States. Although transmission of these infections to other humans may be rare in the United States, human-to-bovine transmission may occur regularly: thousands of cases of bovine *Taenia saginata* cysticercosis occurred immediately before and during the dissemination of MR-DT104. As humans are the only definitive host of the *T. saginata* tapeworm, these cases confirm the large-scale occurrence of human-to-animal transmission of enteropathogenic agents in the United States. Since transmission of *S.* Typhimurium from herd to herd is common in the United States, increased emphasis on *Salmonella* infection control may be an effective method for reducing dissemination of organisms such as MR-DT104.

With or without imposition of stringent controls on antibiotic use in the United States and Europe, the future genetic emergence of new epidemic clones of *S.* Typhimurium somewhere in the world is highly likely, and controlling the dissemination of epidemic clones is essential to avoid increasing problems with multidrug resistance. Certainly we do not disagree with the concept of reducing antimicrobial use, particularly such frivolous use as in calf milk replacers. However, we urge public health officials to consider that infection control is as central to control of agents such as MR-DT104 as it is for epidemic MRSA.

**Margaret A. Davis, Dale D. Hancock, Thomas E. Besser, Daniel H. Rice, John M. Gay**
Washington State University, Pullman, Washington, USA

### References

1. Cohen ML, Tauxe RV. Drug-resistant *Salmonella* in the United States: an epidemiologic perspective. Science 1986;234:964-9.
2. Glynn MK, Bopp C, Dewitt MS, Dabney P, Mokhtar M, Angulo FJ. Emergence of multidrug-resistant *Salmonella enterica* serotype Typhimurium DT104 infections in the United States. N Engl J Med 1998;338:1333-8.
3. Mølbak K, Baggesen DL, Aarestrup FM, Ebbesen JM, Engberg J, Frydendahl K, et al. An outbreak of multidrug-resistant, quinolone-resistant *Salmonella enterica* serotype Typhimurium DT104. N Engl J Med 1999;341:1420-5.
4. Smith KE, Besser JM, Hedberg CW, Leano FT, Bender JB, Wicklund JH, et al. Quinolone-resistant *Campylobacter jejuni* infections in Minnesota, 1992-1998. N Engl J Med 1999;340:1525-32.
5. Papakyriacou H, Vaz D, Simor A, Louie M, McGavin MJ. Molecular analysis of the accessory gene regulator (agr) locus and balance of virulence factor expression in epidemic methicillin-resistant *Staphylococcus aureus*. J Infect Dis 2000;181:990-1000.
6. Wagenvoort JHT. Dutch measures to control MRSA and the expanding European Union. Eurosurveillance 2000;5:26-8.
7. Lipsitch M, Bergstrom CT, Levin BR. The epidemiology of antibiotic resistance in hospitals. Proc Nat Acad Sci U S A 2000;97:1938-43.
8. McGowan JE Jr. Strategies for study of the role of cycling on antimicrobial use and resistance. Infect Control Hosp Epidemiol 2000 Jan;21(1 Suppl):S36-S43.
9. Ng LK, Mulvey MR, Martin I, Peters GA, Johnson W. Genetic characterization of antimicrobial resistance in Canadian isolates of *Salmonella* Serovar Typhimurium DT104. Antimicrob Agents Chemother 1999;43:3018-21.
10. Briggs CE, Fratamico PM. Molecular characterization of an antibiotic resistance gene cluster of *Salmonella typhimurium* DT104. Antimicrob Agents Chemother 1999;43:846-9.
11. Pitt TL, Livermore DM, Miller G, Vatopoulos A, Legakis NJ Resistance mechanisms of multiresistant serotype 012 *Pseudomonas aeruginosa* isolated in Europe. J Antimicrob Chemother 1990;26(3):319-28.

### Malaria and Global Warming in Perspective?

**To the Editor:** The two reports from the International Panel on Climate Change (IPCC) (1,2) cited in the letter by Pim Martens (3) are widely regarded as "the standard scientific reference for all concerned with climate change and its consequences," yet the contents of these reports are often misleading. The quoted passage does not acknowledge the devastation caused by malaria in temperate regions. The reassurance that "existing public health resources" would "make reemergent malaria unlikely" ignores the nonclimatic factors that led to its disappearance and continued absence. Moreover, although malaria/climate models are not meant to predict future worlds, the IPCC chapter (1) on human health—one-third of which is devoted to vector-borne disease—makes extensive use of such models to warn of substantial "actual climate-related increases in malaria incidence" and "highly likely" extensions of its distribution. The chapter does include statements that the "predictions" of such models should be viewed cautiously "until they have been validated against historical data sets," and "malaria is most likely to extend its spread…in tropical countries." The past presence of malaria in "southern Europe" is also mentioned, but such qualifiers are applied to predictions of 10- to 100-fold increases in epidemic potential in temperate climates. These predictions are frequently cited as evidence of a major threat to humanity (4,5).

The IPCC reports state "…anopheline mosquito species that transmit malaria do not usually survive where the mean winter temperature drops below 16°-18°C." Similarly, two oft-quoted publications (6,7) define the vector's limit of survival as the 15°C winter isotherm, i.e., in the northern Sahara. However, in the past the limit was the 15°C summer isotherm. In fact, much of Europe and all of the United States are within the 20°C or 25°C summer isotherms, and malaria was once prevalent in parts of southern Canada and up to 64°N in Russia and Siberia. The same publications state that *Aedes aegypti*, the principal urban vector of dengue and yellow fever, cannot survive mean temperatures below 10°C, but with global warming "…dengue could extend into the southern United States." This statement has been repeatedly quoted (5), although *Ae. aegypti* is common where winter temperatures of -15°C are not unusual and epidemics of dengue and yellow fever have occurred as far north as Boston and Dublin. Repeated claims that global warming may have already led to increases in these diseases in the tropics are equally indefensible (8,9).

**Paul Reiter**
Centers for Disease Control and Prevention, San Juan, Puerto Rico, USA

### References

1. Intergovernmental Panel on Climate Change (IPCC). Impacts, adaptations and mitigation of climate change: scientific-technical analyses. Watson RT, Zinyowera MC, Moss RH, editors. New York: Cambridge University Press;1996. Chapter 18.
2. Intergovernmental Panel on Climate Change. The regional impacts of climate change: an assessment of vulnerability. Working Group II. Intergovernmental Panel on Climate Change. New York: Cambridge University Press; 1998. Chapters 5,8.
3. Martens P. Malaria and global warming in perspective? [letter] Emerg Infect Dis 2000;6:313-4.
4. Ross A. Strange weather: culture, science and technology in the age of limits. London: Verso; 1996.
5. Gelbspan R. The heat is on: the high stakes battle over Earth's threatened climate. New York: Addison-Wesley; 1997.
6. Patz JA, Epstein PR, Burke TA, Balbus JM. Global climate change and emerging infectious diseases. JAMA 1996;275:217-23.
7. Epstein PR, Diaz HF, Elias S, Grabherr G, Graham NE, Martens WJM, et al. Biological and physical signs of climate change: focus on mosquito-borne diseases. Bulletin of the American Meteorological Society 1998;79:409-17.
8. Reiter P. Global warming and vector-borne disease in temperate regions and at high altitude. Lancet 1998;351:839-40.
9. Mouchet J, Manguin S, Sircoulon J, Laventure S, Faye O, Onapa AW, et al. Evolution of malaria in Africa for the past 40 years: impact of climatic and human factors. J Am Mosq Control Assoc 1998;14:121-30.

